# 1065. Efficacy of Cefiderocol in Experimental *Stenotrophomonas maltophilia* Pneumonia in Persistently Neutropenic Rabbits

**DOI:** 10.1093/ofid/ofab466.1259

**Published:** 2021-12-04

**Authors:** Vidmantas Petraitis, Ruta Petraitiene, Povilas Kavaliauskas, Ethan Naing, Andrew Garcia, Naoki Ishibashi, Benjamin Georgiades, Roger Echols, Robert A Bonomo, Yoshinori Yamano, Thomas J Walsh

**Affiliations:** 1 Weill Cornell Medicine of Cornell University, New York, NY; 2 Transplantation–Oncology Infectious Disease Program, Translational Research Laboratory, Division of Infectious Diseases, Weill Cornell Medicine, New York, NY 10065, US, New York, NY; 3 Pharmaceutical Research Division, Osaka, Osaka, Japan; 4 Shionogi, Inc., Florham Park, New Jersey; 5 Infectious Disease Drug Development Consulting LLC, Easton, Connecticut; 6 Louis Stokes Cleveland VA Medical Center, Cleveland, OH; 7 Shionogi & Co., Ltd., Osaka, Osaka, Japan; 8 Weill Cornell Medicine, New York, NY

## Abstract

**Background:**

*Stenotrophomonas maltophilia* causes lethal pneumonia, bacteremia, and sepsis in immunocompromised patients. As a standard of care, trimethoprim-sulfamethoxazole (T/S) is considered to be the first-line therapy for *Stenotrophomonas* pneumonia. Cefiderocol (CFDC) is a new parenteral siderophore cephalosporin that is transported through the outer cell membrane as a siderophore mimic that then inhibits Gram-negative cell wall biosynthesis. CFDC has potent activity *in vitro* against *S. maltophilia*; however, little is known about its *in vivo* activity against *Stenotrophomonas* pneumonia in immunocompromised hosts. We therefore studied CFDC in comparison to TS in the persistently neutropenic rabbit model of *Stenotrophomonas* pneumonia. This rabbit model, in contrast to conventional murine models, reflects the human pattern of infection more accurately over time.

**Methods:**

We initially studied the plasma pharmacokinetics of CFDC in non-infected and infected animals. *Stenotrophomonas* pneumonia was established by direct endotracheal inoculation of *S. maltophilia* 1×10^10^ CFUs for tracheobronchial colonization that evolved into bronchopneumonia. Experimental groups consisted of CFDC, T/S, and untreated controls (UC). Rabbits received CFDC at 120 mg/kg IV Q8h and T/S at 5 mg/kg IV Q12h. Profound persistent neutropenia was maintained with cytosine arabinoside. Treatment was continued for 10 days.

**Results:**

There were no significant differences between non-infected and infected rabbits in CFDC pharmacokinetics. Rabbits treated with CFDC and T/S demonstrated significant decreases of residual pulmonary and BAL bacterial burden vs UC (p≤0.001). CFDC achieved full clearance of *S. maltophilia* from lung tissue and BAL. This antibacterial activity coincided with significant reduction of lung weights (marker of organism-mediated pulmonary injury) in the CFDC group vs T/S and UC (p< 0.01). Survival was prolonged in the CFDC treatment group with 87% survival in comparison to that of T/S (25%) and UC (0%) (p< 0.01).

Table 1. Efficacy of Cefiderocol in Experimental *Stenotrophomonas maltophilia* Pneumonia in Persistency Neutropenic Rabbits

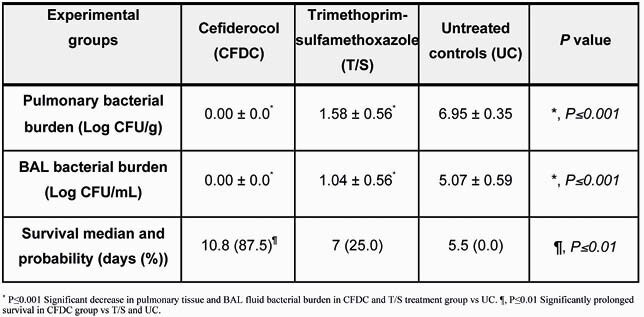

**Conclusion:**

Cefiderocol is highly active in treatment of experimental *S. maltophilia* pneumonia in persistently neutropenic rabbits, thus laying the foundation for future clinical investigations against this lethal infection.

**Disclosures:**

**Naoki Ishibashi, MD**, **Shionogi, Inc.** (Employee) **Benjamin Georgiades, n/a**, **Shionogi, Inc.** (Consultant) **Roger Echols, MD**, **Shionogi** (Consultant) **Robert A. Bonomo, MD**, **entasis** (Research Grant or Support)**Merck** (Grant/Research Support)**NIH** (Grant/Research Support)**VA Merit Award** (Grant/Research Support)**VenatoRx** (Grant/Research Support) **Yoshinori Yamano, PhD**, **Shionogi** (Employee) **Thomas J. Walsh, MD, PhD (hon**), **Scynexis** (Consultant, Grant/Research Support)**Shionogi** (Consultant, Grant/Research Support)

